# Analysis of the Efficacy and Mechanism of Action of Xuebijing Injection on ARDS Using Meta-Analysis and Network Pharmacology

**DOI:** 10.1155/2021/8824059

**Published:** 2021-05-22

**Authors:** Yun Zhang, Jie Wang, Yong-Mei Liu, Hui Yang, Guang-Jun Wu, Xuan-Hui He

**Affiliations:** ^1^Immunology Research Department, Guang'anmen Hospital, China Academy of Chinese Medical Sciences, Beijing, China; ^2^Department of Cardiology, Guang'anmen Hospital, China Academy of Chinese Medical Sciences, Beijing, China

## Abstract

**Objective:**

Acute respiratory distress syndrome (ARDS) is defined as the acute onset of noncardiogenic edema and subsequent gas-exchange impairment due to a severe inflammatory process known as cytokine storm. Xuebijing injection (hereinafter referred to as Xuebijing) is a patent drug that was used to treat ARDS or severe pneumonia (SP) in China. However, its efficacy and mechanism of actions remain unclear. In this study, we used meta-analysis and network pharmacology to assess these traits of Xuebijing.

**Methods:**

We searched PubMed, Embase, Cochrane Library, China National Knowledge Infrastructure (CNKI), and Wanfang databases for randomized controlled trials (RCTs) that evaluated Xuebijing therapy for ARDS or SP. The outcomes were total mortality, intensive care unit (ICU) stay time, and TNF-*α* and IL-6 levels. We performed a meta-analysis using RevMan 5.3 software. The putative targets, top 10 proteins, and possible pathway of Xuebinjing on ARDS were analyzed by network pharmacology. TNF-*α* and IL-6 were further docked with the six main active components of Xuebinjing using AutoDock 4.2.6 and PyMol 1.5.0.3 software.

**Results:**

Fifteen RCTs involving 2778 patients (13 ARDS and 2 SP) were included. Compared with the control, Xuebijing treatment significantly reduced the mortality rate (risk ratio, 0.64 (95% credible interval (CrI), 0.54–0.77)), reduced the ICU stay time (mean difference (MD), -4.51 (95% CrI, -4.97–-4.06)), reduced the TNF-*α* ((MD), -1.23 (95% CrI, -1.38–-1.08)) and IL-6 ((MD), -1.15 (95% CrI, -1.52–-0.78)) levels. The 56 putative targets, top 10 proteins (MAPK1 (mitogen-activated protein kinase 1), MAPK8 (mitogen-activated protein kinase 8), RELA (transcription factor p65), NFKB1 (nuclear factor NF-kappa-B p105 subunit), JUN (transcription factor AP-1), SRC (proto-oncogene tyrosine-protein kinase), TNF (tumor necrosis factor), HRAS (GTPase HRas), IL6 (interleukin-6), and APP (amyloid-beta A4 protein)), and possible pathways (Ret tyrosine kinase, IL2-mediated signaling events, CD4+/CD8+ T cell-related TCR signaling, p75(NTR)-mediated signaling, CXCR4-mediated signaling events, LPA receptor-mediated events, IL12-mediated signaling events, FAS (CD95) signaling pathway, and immune system) of Xuebinjing's action on ARDS were obtained. The molecular docking results showed that all the six components of Xuebinjing docked with TNF-*α*, and two components docked with IL-6 got the binding energies lower than -5.

**Conclusion:**

Our results recommended Xuebijing treatment for patients with ARDS. Xuebijing has therapeutic effects on ARDS patients partly by regulating the immune cell/cytokine pathways and thus inhibiting the cytokine storm. TNF-*α* is the cytokine both directly and indirectly inhibited by Xuebijing, and IL-6 is the cytokine mainly indirectly inhibited by Xuebijing.

## 1. Introduction

Acute respiratory distress syndrome (ARDS) always occurs after a precipitating factor, most frequently of pneumonia, shock, aspiration of gastric contents, sepsis, or trauma [1]. Patients with ARDS exhibit a high mortality rate (50%-60%) because of complications such as sepsis, multiorgan failure, refractory shock, and refractory hypoxemia [2]. Survivors of ARDS patients often suffered from chronic adverse outcomes such as fibrosis, tracheal stenosis, pulmonary function decline, muscle weakness, ambulatory dysfunction, and overall poor quality of life [3, 4].

ARDS is a complex clinical syndrome characterized by acute inflammation, microvascular damage, and increased pulmonary vascular and epithelial permeability [5, 6]. According to the understanding of the pathogenesis of ARDS, the immune system is a very important participant [7]. The levels of serum cytokine and chemokine in ARDS patients correlated with the severity of lung injury [8]. Infected epithelial cells produce cytokines that attract leukocytes, macrophages, and adjacent endothelial cells to further infiltrate and induce a higher level of cytokine and chemokine, and the symptom often called cytokine storm [9]. Although considerable progress has been made in understanding the pathogenesis of ARDS, little progress has been made in the development of specific therapies to combat the inflammatory injury of ARDS. Thus, drugs for the treatment of ARDS, especially the inflammatory injury of ARDS are urgently needed.

Xuebijing injection is a patent drug produced by Tianjin Hongri Pharmaceutical Company (China) that has been used for treating ARDS, SP, sepsis, and MODS [10–13]. Additionally, Xuebijing has recently been approved for treating COVID-19 in China [14, 15]. However, the efficacy of Xuebijing in the treatment of ARDS is not fully understood, and the mechanisms of action remain unclear. In this study, meta-analysis and network pharmacology were used to unveil these traits of Xuebijing on ARDS.

## 2. Materials and Methods

This article was prepared in accordance with the Preferred Reporting Items for Systematic Reviews and Meta-Analyses (PRISMA) guidelines and a previously published protocol (PROSPERO: CRD42020173346).

### 2.1. Search Strategy

A systematic search of MEDLINE, Embase, Central, China National Knowledge Infrastructure (CNKI), and Wanfang databases was performed from inception to April 10, 2021, with no language restriction. Unpublished trials were also identified from clinical trial registry platforms. The reference lists of the included studies were searched manually for additional studies. Randomized controlled trials (RCTs) consisting of medical subject headings (MSH) and free texts with patient relevant terms (ARDS or SP) and intervention relevant terms (Xuebijing injection) were included.

### 2.2. Inclusion and Exclusion Criteria

**Figure 1:**
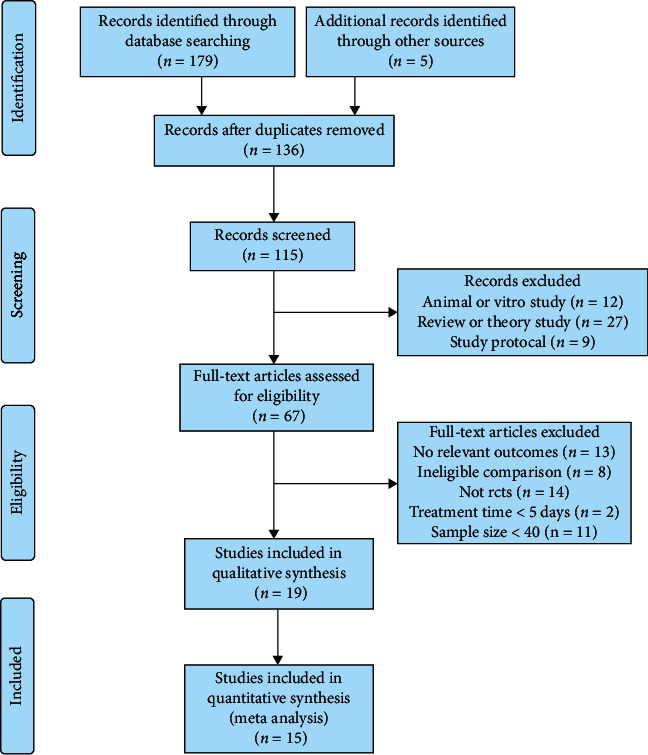
Summary of study retrieval and identification.

The inclusion criteria were as follows: (1) RCTs, (2) Xuebijing treatment versus control, (3) patients with ARDS or SP aged 18 years or older, and (4) parameters of mortality, intensive care unit (ICU) stay, IL-6, or TNF-*α*. The exclusion criteria were as follows: (1) patients with mild or common pneumonia (nonmechanical ventilation or oxygenation index PaO_2_/FiO_2_ ≥ 300 mmHg according to the exclusion criteria for ARDS in Berlin 2012 [16]); (2) lacking literature data (e.g., nonpaired studies); (3) study design, not RCT; (4) duplicate publications; (5) conference reports, system reviews, protocols, or abstracts; and (6) RCTs with small sample sizes (*n* < 40; Figure 1).

### 2.3. Data Extraction and Quality Assessment

After the removal of duplicates, the titles and abstracts of search results were screened for relevance by a single author (Y. Zhang or YM. Liu). The full texts of the remaining results were independently assessed in duplicate by two authors (Y. Zhang and H. Yang) for inclusion. The final list of included studies was decided based on a discussion between the authors with full agreement. Data were extracted using a unified data collection form independently and in duplicate by two authors (Y. Zhang and XH. He). The data extracted from each report included study characteristics (author, year of publication, random method, and sample size), population characteristics (age, sex), intervention characteristics (intervention dosage, duration), and outcomes. RCTs were evaluated by researchers (Y. Zhang, H. Yang, and G.J. Wu) based on the Cochrane risk bias assessment tool [17]. The tool included six domains: random allocation, allocation concealment, blind method, missing outcome data, selecting result report, and other bias. The assessment included assigning a judgment of yes, no, or unclear for each domain to classify as a low, high, or unclear risk of bias, separately. The study was deemed as having a low risk of bias if less than one domain was assumed as unclear or no. If more than four domains were regarded as unclear or no, the study was deemed as having a high risk of bias. The study was regarded as having a moderate risk of bias if two or three domains were considered no or unclear [18]. Review Manager 5.3 (Cochrane Collaboration, Oxford, UK) was used to carry out the quality assessment and investigation of publication bias.

### 2.4. Data Synthesis and Statistical Analysis

Dichotomous data were reported as risk ratios (RRs) with 95% confidence intervals (CIs). Analyses were performed using Markov-chain Monte Carlo methods. Continuous data were calculated as the standard mean difference (SMD) or mean difference (MD) with associated 95% CIs using Cohen's method. Consistency (*I*^2^) was measured for each meta-analysis, and *I*^2^ < 50% was considered with low heterogeneity.

### 2.5. Network Analysis

The main active components of Xuebijing were retrieved from the database and literature. Safflower yellow, danshensu, ligustrazine, paeoniflorin, ferulic acid, and protocatechualdehyde were identified based on quantitative analysis of bioactive constituents using ultrahigh-performance liquid chromatography coupled with high-resolution hybrid quadruple-orbitrap mass spectrometry (UPLC-LCMS) [19]. Danshensu, protocatechualdehyde, paeoniflorin, and safflor yellow A were identified as potential anti-inflammatory components based on a bioactivity-integrated UPLC-Q/TOF assay system [20]. Finally, by verifying on the TCMSP database (https://old.tcmsp-e.com/tcmsp.php), the six active components: safflower yellow A, danshensu, ligustrazine, paeoniflorin, ferulic acid, and protocatechualdehyde, were considered main active components of Xuebijing.

The targets of the six active components of Xuebijing were searched from Pubchem (https://pubchem.ncbi.nlm.nih.gov/), STITCH (http://stitch.embl.de/), SwissTargetPrediction (http://www.swisstargetprediction.ch/), and SEA (http://sea.bkslab.org/) databases. The genes related to ARDS were searched from the Therapeutic Target Database (https://db.idrblab.org/ttd/), DisGeNET (http://www.disgenet. org/search), and Genecards (https://www.genecards.org/) [21]. The putative targets of Xuebijing on ARDS were obtained by overlapping the targets of Xuebijing and the ARDS-related genes and visualized by Cytoscape 3.2.1 software.

The putative targets of Xuebijing on ARDS were further analyzed with internal interaction by String (https://string-db.org/), and the score > 0.9 key node network was constructed by Cytoscape 3.2.1. The top 10 proteins and related genes were selected by cytoHubba, a plugin of Cytoscape [22]. The possible pathways were analyzed by FunRich software (version 3.0) [23].

### 2.6. Molecular Docking

The structures of the six active components of Xuebijing: safflower yellow A, danshensu, ligustrazine, paeoniflorin, ferulic acid, and protocatechualdehyde, and proteins (TNF-*α* and IL-6), were obtained from the databases of PubChem and Protein Data Bank (PDB), respectively. Molecular docking was performed using Autodock version 4.2.6 software (Sousa, Fernandes & Ramos), based on the Lamarckian genetic algorithm, which combines energy evaluation through grids of affinity potential to find a suitable binding position for a ligand on a given protein [24]. All hydrogen atoms were added to the protein targets, and Kollman united atomic charges were computed. The grid box was allocated properly to include the active residue in the center. The genetic algorithm and its run were set to 1000, as the docking algorithms were set on default. Finally, results were retrieved as binding energies, and dockings with binding energies lower than -5 were selected as significant binding events and were visualized using PyMol version 1.5.0.3. software [25].

## 3. Results

### 3.1. The Efficacy of Xuebijing on ARDS

#### 3.1.1. Overall Characteristics of Studies

In total, 15 RCTs (13 ARDS and 2 SP) involving 2778 patients were included [20–28]. The male participants were (1140; 41.03%), and the mean age ranged from 38.6 to 65 years. The detailed demographic and clinical characteristics of the included studies are shown in Table 1.

#### 3.1.2. Risk of Bias and Publication Bias

**Figure 2:**
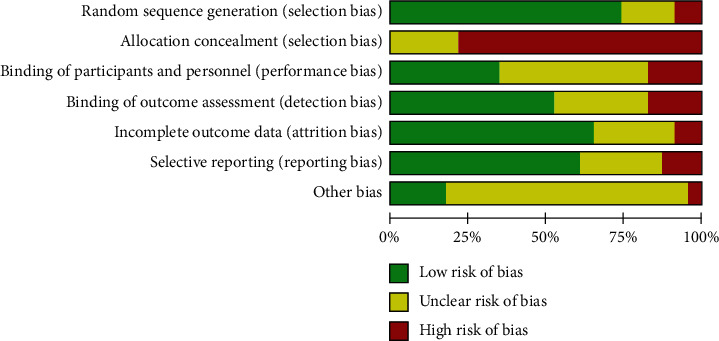
Risk of bias graph for all the included studies.

Of the 15 included studies, five (33.3%) had a low risk of bias, six (40%) had a high risk of bias, and four (26.7%) had a moderate risk of bias (Figure 2).

#### 3.1.3. Total Mortality

**Figure 3:**
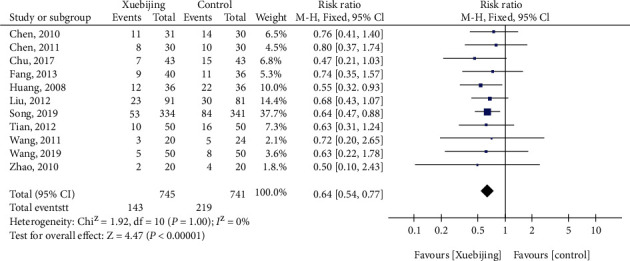
Forest plot of Xuebijing treatment versus control for total mortality.

For total mortality, eleven studies had enrolled 1486 patients with ARDS or SP. There were 305 deaths, including 143 (19.19%) of 745 participants treated with Xuebijing and 219 (29.55%) of 741 patients in the control groups. Compared with the control groups, Xuebijing treatment (RR, 0.64 (95% credible interval (CrI), 0.54–0.77)) was associated with reduced mortality rate (Figures 3).

#### 3.1.4. ICU Stay Time

**Figure 4:**
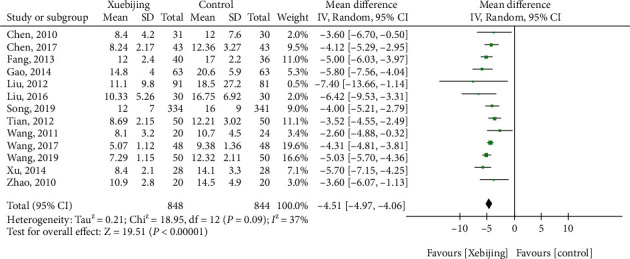
Forest plot of Xuebijing treatment versus control for ICU stay.

For ICU stay time, thirteen studies had enrolled 1692 patients with ARDS or SP. Compared with the control groups, Xuebijing treatment (MD, -4.51 (95% CrI, -4.97–-4.06)) was associated with reduced ICU stay time (days) in the hospital (Figure 4).

#### 3.1.5. TNF-*α* Level

**Figure 5:**
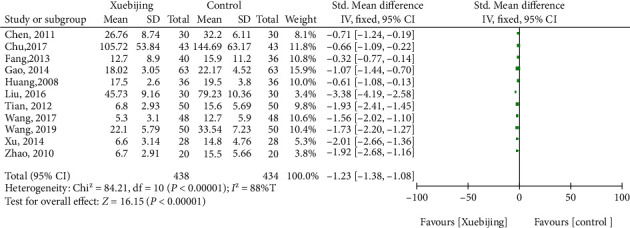
Forest plot of Xuebijing treatment versus control for TNF-*α* level.

For the outcome of TNF-*α*, eleven studies had enrolled 872 ARDS patients. Compared with the control groups, Xuebijing treatment (SMD, -1.23 (95% CrI, -1.38 to -1.08)) were associated with decreased the TNF-*α* levels (Figure 5.).

#### 3.1.6. IL-6 Level

**Figure 6:**
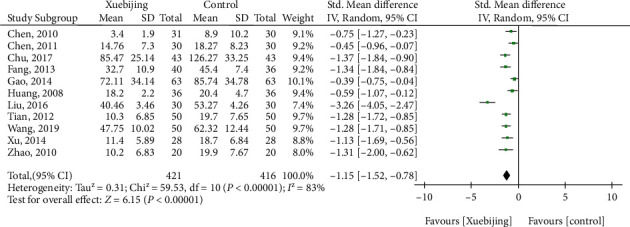
Forest plot of Xuebijing treatment versus control for IL-6 level.

For the outcome of IL-6 levels, eleven studies had enrolled 837 ARDS patients. Compared with the control groups, Xuebijing treatment (SMD, -1.15 (95% CrI, -1.52 to -0.78)) were associated with decreased the IL-6 levels (Figure 6.).

#### 3.1.7. Safety

No serious adverse effects of Xuebijing treatment were reported among the included studies.

### 3.2. The Mechanism of Xuebijing on ARDS

#### 3.2.1. The Six Main Active Components of Xuebijing

The six main active components of Xuebijing: safflower yellow A, danshensu, ligustrazine, paeoniflorin, ferulic acid, and protocatechualdehyde, were retrieved as the main active components of Xuebijing (Table 2).

#### 3.2.2. The 56 Putative Targets of Xuebijing on ARDS

**Figure 7:**
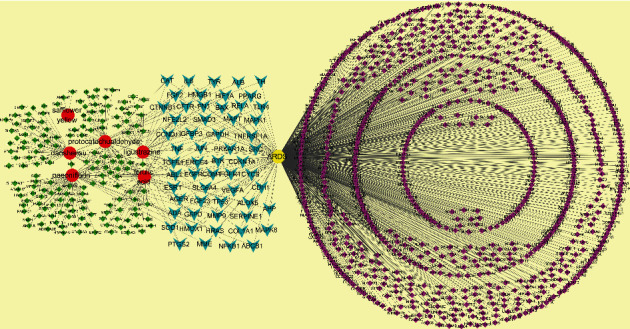
The active components-targets-ARDS-genes network of Xuebijing on ARDS. Note: the network has 1047 nodes, 1086818 shortest paths (99%). The red round node represents active components of Xuebijing. The yellow node represents ARDS. The green node represents the targets of the active components. The pink node represents ARDS-related genes. The blackish green node represents the 56 putative targets of Xuebijing on ARDS.

The 573 targets of the six active components of Xuebijing and 885 genes related to ARDS were obtained. Danshensu had 47, ferulic acid had 54, ligustrazine had 29, paeoniflorin had 69, and protocatechualdehyde had 47 targets. Among them, ferulic acid and ligustrazine shared 5 targets (TNF, MAPK3, MAPK1, PTGS2, and CFTR), and ferulic acid and protocatechualdehyde shared 9 targets (TYR, GLO1, G6PD, CA1, CA5B, sssIM, CA2, CA9, and CA12) (Fig S1). The 56 putative targets of Xuebijing on ARDS were obtained by overlapping (Fig S2). The active components-targets-ARDS-genes network of Xuebijing on ARDS were constructed by Cytoscape 3.2.1. (Figure 7).

#### 3.2.3. The Top 10 Proteins Related with Xuebijing's Action on ARDS

**Figure 8:**
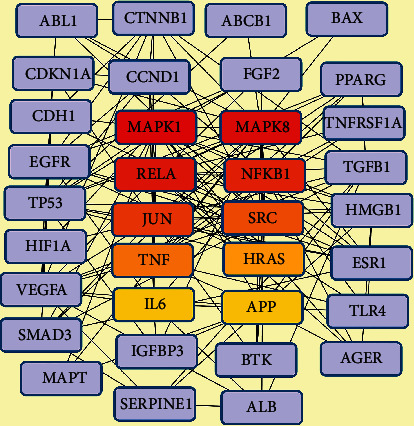
The top 10 proteins related with Xuebijing's action on ARDS. Note: the network has 35 nodes, shortest paths of 1190 (100%). The node color was continuously expressed from red to yellow according to the MCC score high to low. The pink node represents the related genes of the top 10 proteins.

The top 10 proteins (MAPK1, MAPK8, RELA, NFKB1, JUN, SRC, TNF, HRAS, IL6, and APP) related to Xuebijing's action on ARDS were obtained according to the 56 putative targets internal interaction network (Fig S3). The MCC score of MAPK1, MAPK8, RELA, NFKB1, JUN, SRC, TNF, HRAS, IL6, and APP are 1504, 1346, 1344, 1318, 1270, 1228, 1210, 1150, 1124, and 1124, respectively (Figure 8).

#### 3.2.4. The Possible Pathway of Xuebijing's Action on ARDS

**Figure 9:**
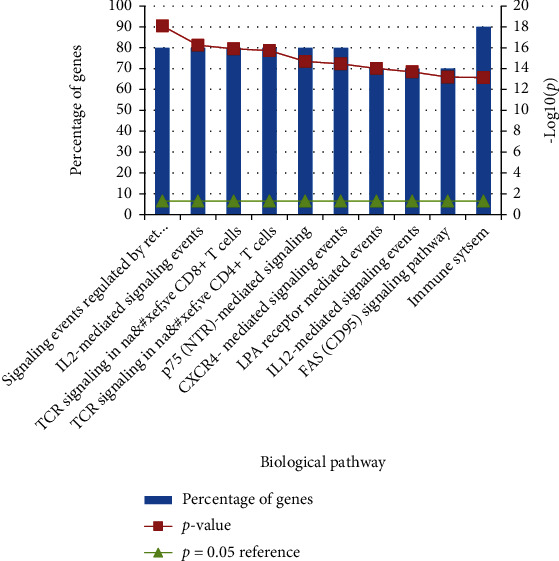
The possible pathway of Xuebijing's action on ARDS.

Based on the top 10 proteins, the possible pathways of Xuebijing's action on ARDS were obtained. They are Ret tyrosine kinase signaling events, IL2-mediated signaling events, CD4+/CD8+ T cells-related TCR signaling, p75(NTR)-mediated signaling, CXCR4-mediated signaling events, LPA receptor-mediated events, IL12-mediated signaling events, FAS (CD95) signaling pathway, and immune system (*P* < 0.05, Figure 9).

#### 3.2.5. Molecular Docking of the Active Components of Xuebijing with TNF-*α* and IL-6

**Figure 10:**
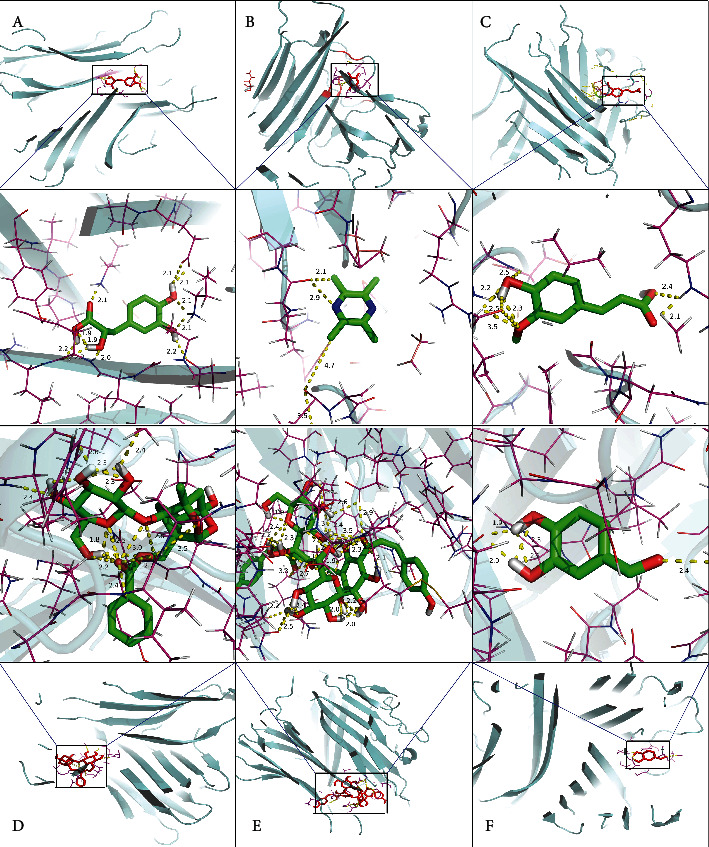
Interaction profiles of the six components docked with TNF-*α*. Note: the six rectangle areas of docked sites were enlarged. (a) Danshensu docked with TNF-*α* (-8.43); (b), ligustrazine docked with TNF-*α* (-6.75); (c), ferulic acid docked with TNF-*α* (-6.68); (d), paeoniflorin docked with TNF-*α* (-6.32); (e), safflor yellow A docked with TNF-*α* (-6.27); (f), protocatechualdehyde docked with TNF-*α* (-6.07). The sticks represent components of Xuebijing, the cartoons represent the secondary structure of the protein, the pink lines represent surrounding residents, the yellow dotted lines represent polar contacts, and the labels are the lengths of the contacts.

The six active components of Xuebijing were further docked with TNF-*α* and IL-6. The molecular structure of those components and proteins was exhibited in the supplemental material (Fig S4). The results showed that the six components with TNF-*α* and two components with IL-6 got binding energies lower than -5 kcal/mol. Danshensu had the strongest interaction with TNF-*α* (-8.43 kcal/mol; Table 3). The interaction profiles of the six components docked with TNF-*α* are shown in Figure 10.

## 4. Discussion

In ARDS patients, an inflammatory response known as cytokine storm could lead to multiple organ failure and invariably fatal [40]. Cytokine storm involves activation and release of inflammatory cytokines such as interleukin (IL), tumor necrosis factor (TNF), interferon (IFN), and C-X-C motif chemokine (CXCL), in a positive feedback loop of pathogen-triggered inflammation [41]. With the exudation of inflammatory factors, cytokines increase abnormally in other tissues and organs, interfering with the immune system, causing the excessive immune response of the body, resulting in diffuse damage of lung cells, pulmonary fibrosis, and multiple organs [42].

Currently, there is no proven treatment to combat this systemic response of cytokine storm. Future progress will depend on finding therapeutics that inhibit cytokine storm, thereby alleviating lung and multiorgan damages. In this study, Xuebijing showed significant efficacy for treating ARDS patients through meta-analysis. Xuebijing treatment reduced the mortality rate, decreased the ICU stay time, and reduced IL-6 and TNF-*α* levels when compared with the control groups.

To further study the mechanism of Xuebijing treatment on ARDS, we used the network pharmacology analysis. The top 10 proteins of Xuebijing's action on ARDS were MAPK1, MAPK8, RELA, NFKB1, JUN, SRC, TNF, HRAS, and IL6. The possible pathways of Xuebijing's action on ARDS were mainly related with immune cell/cytokine regulating pathways, such as CD4+/CD8+ T cells-related TCR signaling, IL2/IL12-mediated signaling events, p75(NTR)-mediated signaling, CXCR4-mediated signaling events, FAS (CD95) signaling pathway, LPA receptor-mediated events, and immune system.

The generation of CD4+ and CD8+ T cell lineages from CD4+ CD8+ double-positive (DP) thymocyte precursors is a complex process initiated by engagement of major histocompatibility complex (MHC) and T cell receptor (TCR) [43]. CD4+ T helper (Th) lymphocytes are divided into Th1 and Th2 based on their profile of cytokine production. Th1 cells, which produce IFN-gamma, IL-2, and TNF, evoke cell-mediated immunity and phagocyte-dependent inflammation. An imbalance of CD4+/CD8+ T cell can lead to a hyperinflammatory condition (cytokine storm), leading to compensatory anti-inflammatory response syndrome (CARS), and, above all, an immune paralysis stat [44]. p75 neurotrophin receptor (p75(NTR), or aka CD271) signaling is expressed by certain innate immune cells. p75(NTR) was upregulated upon infection and affected innate immune cell behavior to producing the cytokines: IL-10, IL-6, and IL-1*α* [45]. The CXCR4 is a receptor of its natural ligand (chemokine CXCL12). Activation of CXCR4 results in a long chain of intracellular and extracellular events, including the release of the proinflammatory cytokine TNF-*α* and prostanglandins [46]. FAS (CD95) is a member of the TNF receptor superfamily. FAS are known to negatively regulate LPS-induced proinflammatory responses and reducing the production of TNF-*α*, IL-8, IL-6, and IL-12, implicated in immune homeostasis and immune surveillance [47, 48]. Under acute and chronic inflammatory conditions, LPA concentration was significantly increased and induced the secretion of IL-6, TNF-*α*, IL-1*β*, CXCL10, CXCL2, and CCL5 [49].

Based on these results, we deduced that Xuebijing has therapeutic effects on patients with ARDS partly by regulating the immune cell/cytokine pathways, such as maintaining CD4+ and CD8+ T cell balance, inhibiting p75(NTR) signaling, inhibiting CXCR4 and LPA signaling, promoting FAS (CD95) signaling, and thus inhibiting the producing and releasing of cytokines and chemokines.

IL-6 and TNF-*α* were believed as the pivotal cytokines related to cytokine storm [50]. We docked the six main active components of Xuebijing with IL-6 and TNF-*α*. The results showed that the six components with TNF-*α* and two components with IL-6 got significant binding energies. We deduced that TNF-*α* is the cytokine both directly and indirectly inhibited by Xuebijing, and IL-6 is the cytokine mainly indirectly inhibited by Xuebijing.

## 5. Conclusion

In conclusion, our results supported the use of Xuebijing treatment for patients with ARDS. Additionally, we found that Xuebijing has therapeutic effects on patients with ARDS partly by regulating the immune cell/cytokine pathways. TNF-*α* is the cytokine both directly and indirectly inhibited by Xuebijing, and IL-6 is the cytokine mainly indirectly inhibited by Xuebijing.

## Figures and Tables

**Table 1:** Demographic and clinical characteristics of the included studies.

Author, year	Random method	Sample size (I/C)	Male (I/C)	Age (years) (I/C)	Intervention	Control	Outcomes	Indications
Fang and Wang, 2013 [26]	Random number table	40/36	22/19	52.4 ± 13.7/51.5 ± 13.3	Xuebijing 100 mL iv q12h for 7 days	Conventional treatment	Mortality, ICU stay, IL-6, TNF-*α*,	ARDS
Liu et al., 2012	Random number table	91/81	68/57	55/53	Xuebijing 100 mL iv q12h for 7 days	Conventional treatment	Mortality, ICU stay	ARDS
Song et al., 2019 [27]	Random number	334/341	224/234	58.67 ± 13.58/58.13 ± 14.24	Xuebijing 100 mL iv q12h for 7 days	Placebo (saline 100 mL)	Mortality, ICU stay	SP
Chu, 2017 [28]	Draw lots	43/43	25/26	47.63 ± 21.69/47.35 ± 22.07	Xuebijing 100 mL iv q12h for 7 days	Low molecular weight heparin 5 U/kg/h)	Mortality, ICU stay, IL-6, TNF-*α*	ARDS
Gao et al., 2014 [29]	Random number	63/63	34/36	50.22 ± 18.86/51.02 ± 19.19	Xuebijing 100 mL iv bid for 7 days	Conventional treatment	ICU stay, IL-6, TNF-*α*	SP
Huang et al., 2008 [30]	Random number	36/36	20/22	46 ± 16/44 ± 13	Xuebijing 50 mL iv bid for 7 days	Conventional treatment	Mortality, IL-6, TNF-*α*	ARDS
Tian and Sun, 2012 [31]	Draw lots	50/50	57	39.5 ± 3.7	Xuebijing 50 mL iv q12h for 7 days	Conventional treatment	Mortality, ICU stay, IL-6, TNF-*α*	ARDS
Liu et al., 2016 [32]	Random number table	30/30	17/16	54.3 ± 5.9/53.8 ± 6.2	Xuebijing 50 mL iv bid for 7 days	Conventional treatment	ICU stay, IL-6, TNF-*α*	ARDS
Wang, 2017 [33]	Random number table	48/48	26/25	38.6 ± 5.8/39.1 ± 5.4	Xuebijing 50 mL iv bid for 7 days	Conventional treatment	ICU stay, TNF-*α*	ARDS
Wang, 2019 [34]	NR	50/50	33/32	62.11 ± 6.21/61.22 ± 7.2	Xuebijing 50 mL iv bid for 7 days	Conventional treatment	Mortality, ICU stay, IL-6, TNF-*α*	ARDS
Chen et al., 2010 [35]	Random number table	31/30	14/16	55.7 ± 17.4/55.7 ± 13.9	Xuebijing 100 mL iv bid for 7 days	Conventional treatment	Mortality, ICU stay, IL-6	ARDS
Xu et al., 2014 [36]	NR	28/28	19/17	44.3 ± 6.5/42.8 ± 5.3	Xuebijing 50 mL iv bid for 7 days	Conventional treatment	ICU stay, IL-6, TNF-*α*	ARDS
Zhao et al., 2010 [37]	NR	20/20	24	41.1 ± 7.31	Xuebijing 50 mL iv bid for 7 days	Conventional treatment	Mortality, ICU stay, IL-6, TNF-*α*	ARDS
Chen and Li, 2011 [38]	Random number table	30/30	33	64 ± 3.5	Xuebijing 100 mL iv qd for 7 days	Conventional treatment	Mortality, IL-6, TNF-*α*	ARDS
Wang et al., 2011 [39]	NR	20/24	12/12	53.6 ± 17.8	Xuebijing 100 mL iv bid for 7 days	Conventional treatment	Mortality, ICU stay	ARDS

Notes: I/C: intervention/control; NR: not reported; SP: severe pneumonia; ARDS: acute respiratory distress syndrome.

**Table 2:** The main active components of Xuebijing.

Main active components of Xuebijing	Canonical SMILES
Safflower yellow	C1 = CC(=CC=C1C=CC(=O)C2 = C(C(C(=C(C2 = O)C=C3C(=O)C(=C(C(C3 = O)(C4C(C(C(C(O4)CO)O)O)O)O)O)C(=O)C=CC5 = CC=C(C=C5)O)O)(C6C(C(C(C(O6)CO)O)O)O)O)O)O
Danshensu	C1 = CC(=C(C=C1CC(C(=O)O)O)O)O
Ligustrazine	CC1 = C(N=C(C(=N1)C)C)C
Paeoniflorin	CC12CC3(C4CC1(C4(C(O2)O3)COC(=O)C5 = CC=CC=C5)OC6C(C(C(C(O6)CO)O)O)O)O
Ferulic acid	COC1 = C(C=CC(=C1)C=CC(=O)O)O
Protocatechualdehyde	C1 = CC(=C(C=C1C=O)O)O

**Table 3:** The binding energy of molecular docking (kcal/mol).

Protein (PBD ID)	Safflor yellow A	Danshensu	Ligustrazine	Paeoniflorin	Ferulic acid	Protocatechualdehyde
TNF-*α* (6OP0)	-6.27	-8.43	-6.75	-6.32	-6.68	-6.07
IL-6 (4J4L)	2.14	-3.34	-3.88	-3.03	-6.1	-5.49

## Abbreviations

ARDS: Acute respiratory distress syndrome
SP: Severe pneumonia
RCTs: Randomized controlled trials
MSH: Medical subject headings
MAPK1: Mitogen-activated protein kinase 1
MAPK8: Mitogen-activated protein kinase 8
RELA: Transcription factor p65
NFKB1: Nuclear factor NF-kappa-B p105 subunit
JUN: Transcription factor AP-1
SRC: Proto-oncogene tyrosine-protein kinase
TNF: Tumor necrosis factor
HRAS: GTPase HRas
IL6: Interleukin-6
APP: Amyloid-beta A4 protein
TCR: T cell antigen receptor
p75(NTR): p75 neurotensin receptor
CXCR4: C-X-C chemokine receptor type 4
LPA: Lysophosphatidic acid
FAS (CD95): Tumor necrosis factor receptor superfamily member 6.

## Additional Points

*Strengths and limitations of this study*. Patients with severe acute respiratory distress syndrome (ARDS) present a formidable treatment challenge. The high mortality and disabling complications of ARDS highlight the need for research on treating ARDS. Xuebijing treatment significantly reduced mortality rates, ICU stay time, IL-6, and TNF-*α* levels. Overall, our findings make a significant contribution to the literature because they support the use of Xuebijing treatment for patients with ARDS. Additionally, through network analysis, we found that there are 56 putative targets, top 10 proteins, and immune cell/cytokine regulation pathways of Xuebijing's actions on ARDS. TNF-*α* is the cytokine both directly and indirectly inhibited by Xuebijing, and IL-6 is the cytokine mainly indirectly inhibited by Xuebijing. One limit of this study was the high heterogeneity with regard to the outcome of TNF-*α* and IL-6 (*I*^2^ > 80%). Additionally, our analysis lacked high-quality RCTs, particularly those with large sample sizes. Thus, further analyses may be required to confirm the possible benefits of Xuebijing for treating ARDS. The other limit was the mechanism of Xuebijing on ARDS mainly based on review and network analysis, lacking experimental validation.

## Supplementary Materials

Supplementary Materials Figure S1: the shared and specific targets of the active components of Xuebijing Danshensu had 47, ferulic acid had 54, ligustrazine had 29, paeoniflorin had 69, and protocatechualdehyde had 47 targets. Among them, ferulic acid and ligustrazine shared 5 targets, and ferulic acid and protocatechualdehyde shared 9 targets. Figure S2: the overlapping map of the components targets and ARDS-related gene C targets represent the targets of the active components of Xuebijing; ARDS DE genes represent ARDS differential expressed genes; the 56 putative targets were obtained as Xuebijing's action on ARDS. Figure S3: the internal interaction network of the 56 putative targets. Figure S4: the molecular structure of the six components and two proteins.
