# Supplementary material for: Analysis of the Efficacy and Mechanism of Action of Xuebijing Injection on ARDS Using Meta-Analysis and Network Pharmacology
Source: Biomed Res Int. 2021 May 22;2021:8824059. doi: 10.1155/2021/8824059 (PMC8166476; doi:10.1155/2021/8824059)
Supplement: Supplementary Materials — Figure S1: the shared and specific targets of the active components of Xuebijing Danshensu had 47, ferulic acid had 54, ligustrazine had 29, paeoniflorin had 69, and protocatechualdehyde had 47 targets. Among them, ferulic acid and ligustrazine shared 5 targets, and ferulic acid and protocatechualdehyde shared 9 targets. Figure S2: the overlapping map of the components targets and ARDS-related gene C targets represent the targets of the active components of Xuebijing; ARDS DE genes represent ARDS differential expressed genes; the 56 putative targets were obtained as Xuebijing's action on ARDS. Figure S3: the internal interaction network of the 56 putative targets. Figure S4: the molecular structure of the six components and two proteins. [file 8824059.f1.docx]

**Description for the supplementary materials**


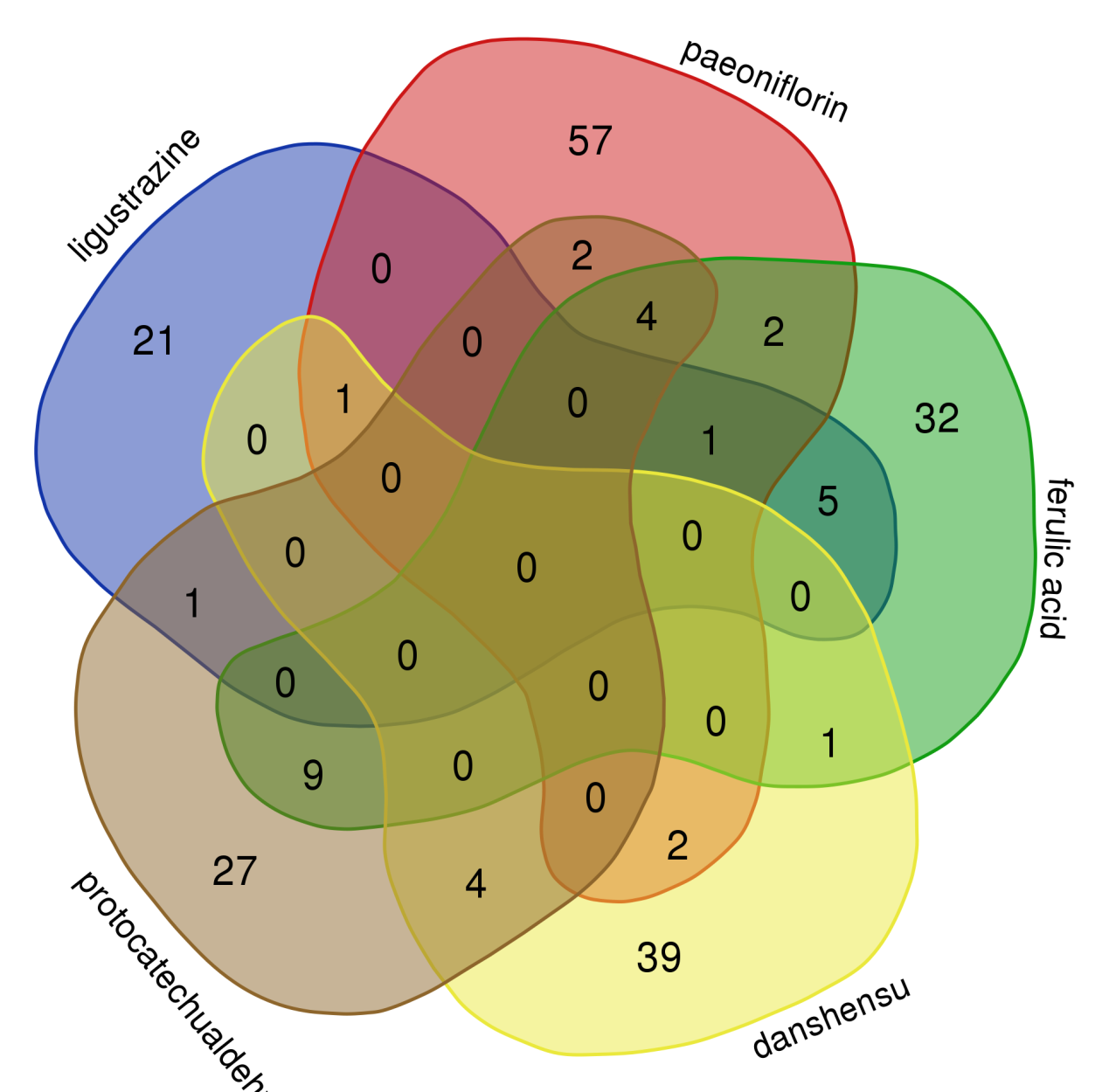


**Fig S1 The shared and specific targets of the active components of Xuebijing**

Danshensu had 47, ferulic acid had 54 , ligustrazine had 29, paeoniflorin had 69, and protocatechualdehyde had 47 targets. Among them, ferulic acid and ligustrazine shared 5 targets, ferulic acid and protocatechualdehyde shared 9 targets.


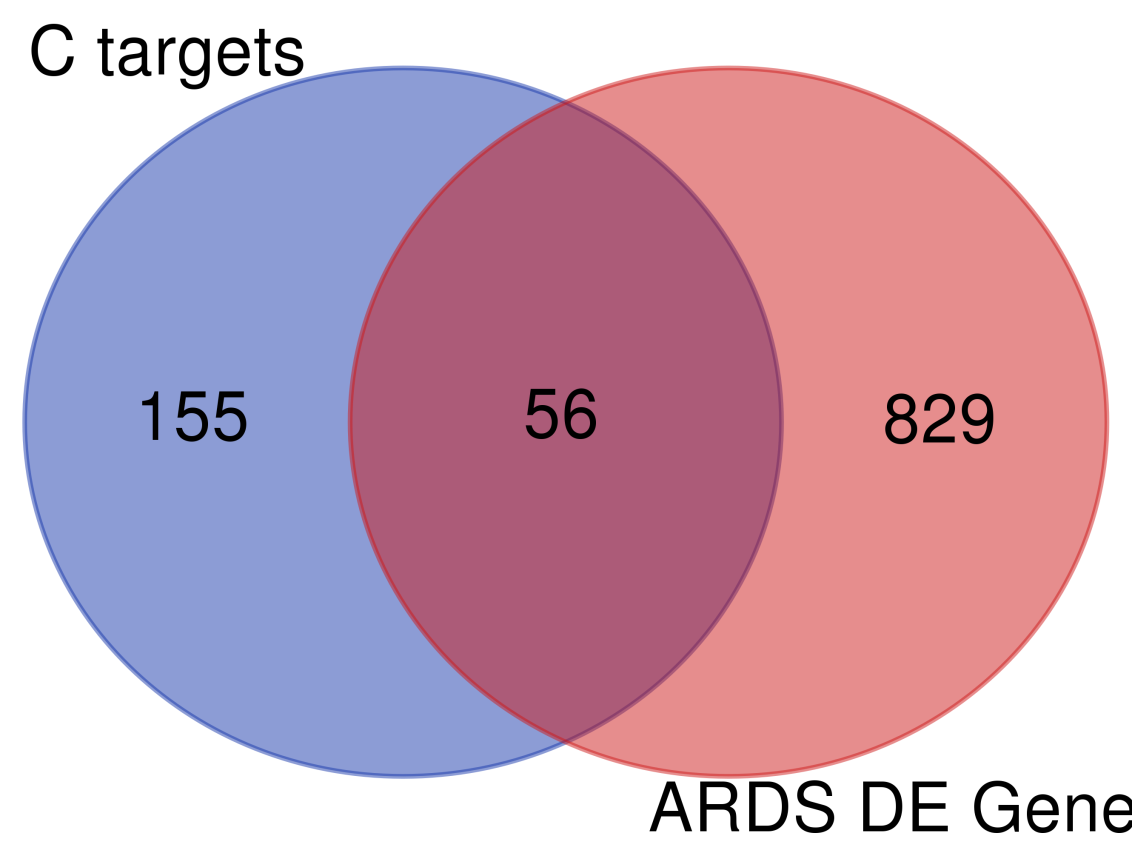


**Fig S2 The overlapping map of the components targets and ARDS related genes**

C targets represent the targets of the active components of Xuebijing; ARDS DE Genes represent ARDS differential expressed genes; the 56 putative targets were obtained as Xuebijing's action on ARDS.


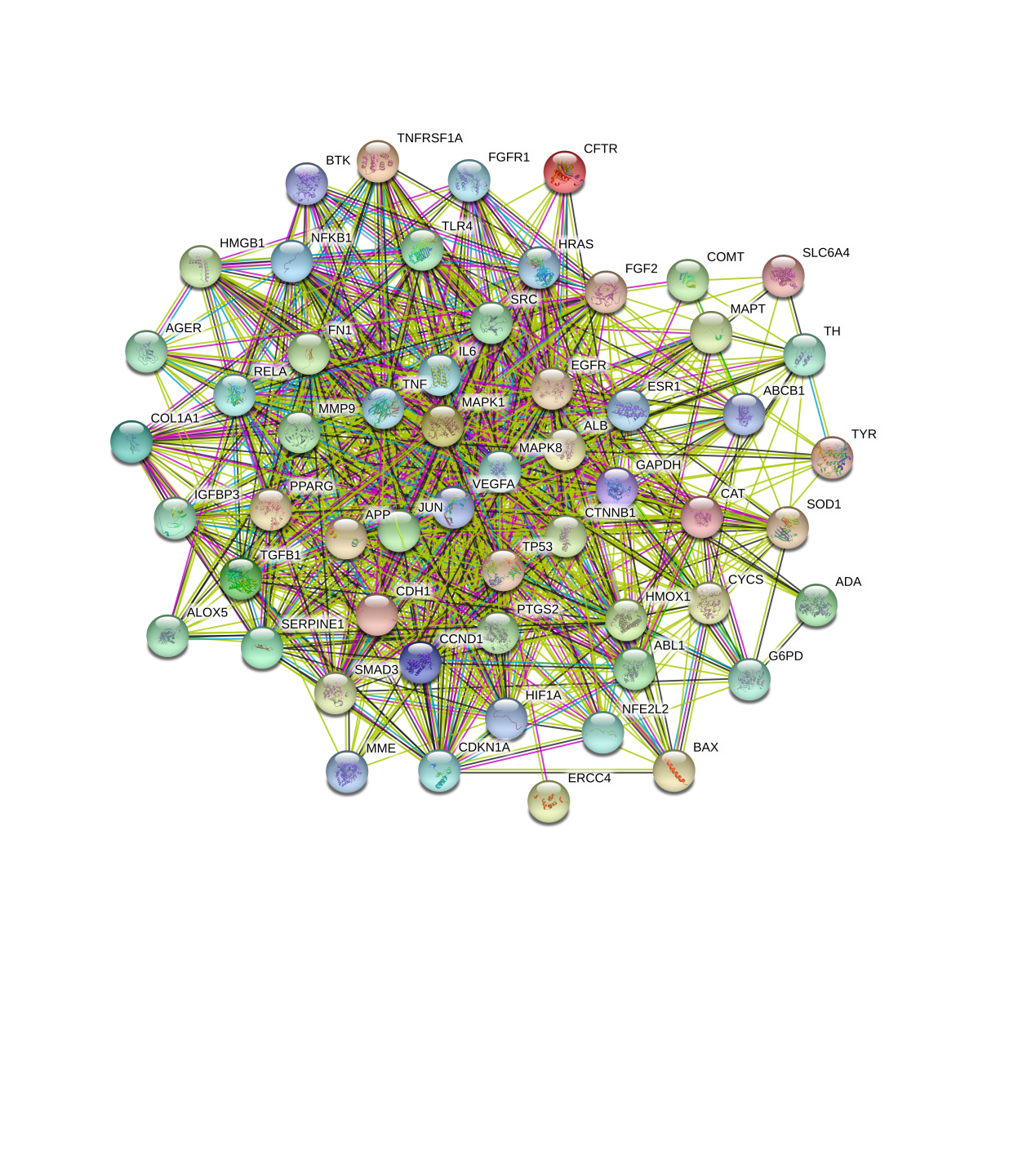


**Fig S3 The internal interaction network of the 56 putative targets**


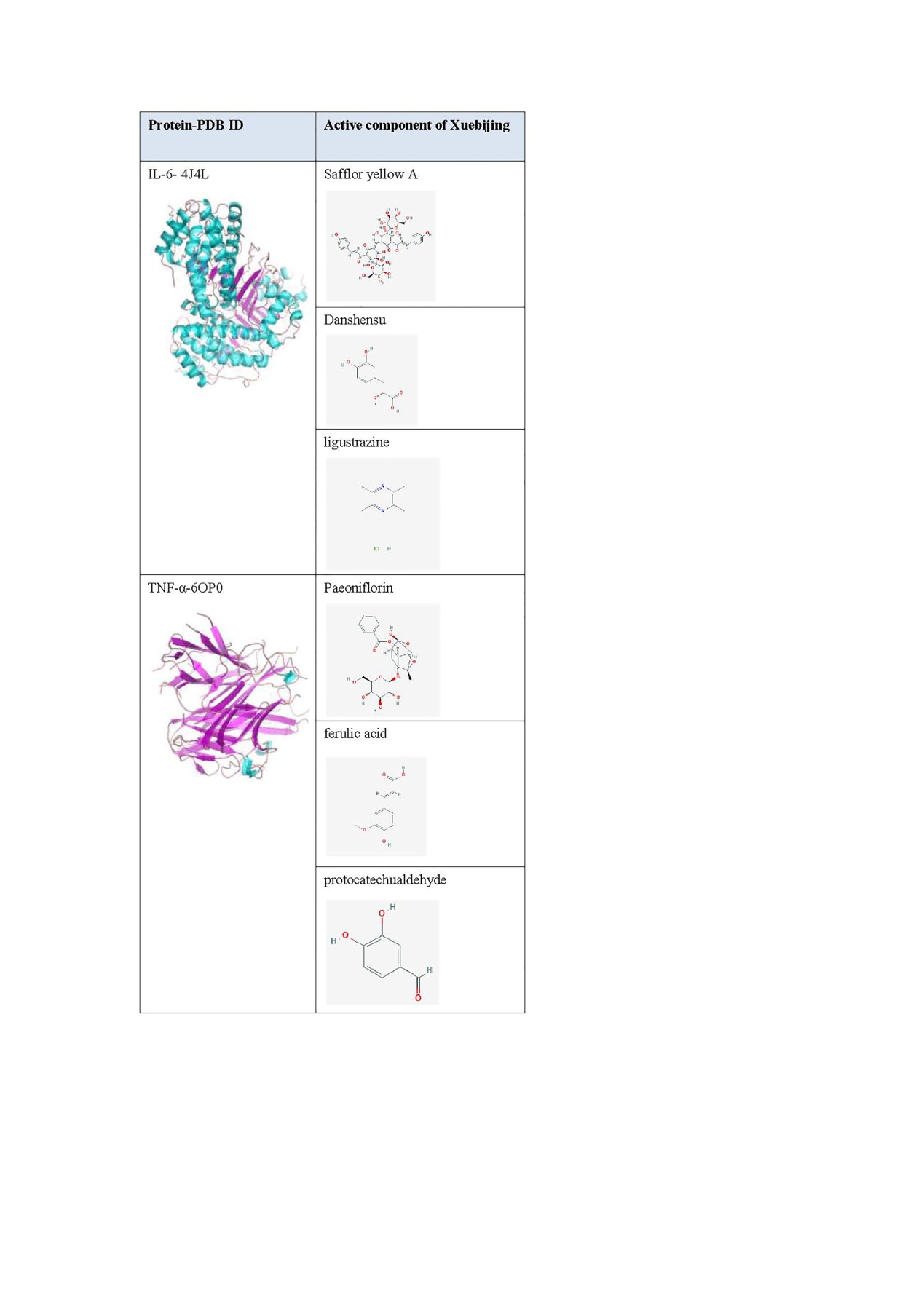


**Fig S4 The molecular structure of the six components and two proteins**
